# B-Vitamin Sharing Promotes Stability of Gut Microbial Communities

**DOI:** 10.3389/fmicb.2019.01485

**Published:** 2019-07-02

**Authors:** Vandana Sharma, Dmitry A. Rodionov, Semen A. Leyn, David Tran, Stanislav N. Iablokov, Hua Ding, Daniel A. Peterson, Andrei L. Osterman, Scott N. Peterson

**Affiliations:** ^1^Infectious and Inflammatory Disease Center, Sanford Burnham Prebys Medical Discovery Institute, La Jolla, CA, United States; ^2^A.A. Kharkevich Institute for Information Transmission Problems, Russian Academy of Sciences, Moscow, Russia; ^3^P.G. Demidov Yaroslavl State University, Yaroslavl, Russia; ^4^Department of Pathology, The Johns Hopkins University School of Medicine, Baltimore, MD, United States

**Keywords:** gut microbiota, B-vitamins, syntrophy, genome reconstruction, community stability

## Abstract

Cross-feeding on intermediary and end-point metabolites plays an important role in the dynamic interactions of host-associated microbial communities. While gut microbiota possess inherent resilience to perturbation, variations in the intake of certain nutrients may lead to changes in the community composition with potential consequences on host physiology. Syntrophic relationships and mutualism at the level of major carbon and energy sources have been documented, however, relatively little is known about metabolic interactions involving micronutrients, such as B-vitamins, biosynthetic precursors of essential cofactors in the mammalian host and numerous members of the gut microbiota alike. *In silico* genomic reconstruction and prediction of community-wide metabolic phenotypes for eight major B-vitamins (B1, B2, B3, B5, B6, B7, B9, and B12), suggests that a significant fraction of microbial gut communities (>20% by abundance) are represented by auxotrophic species whose viability is strictly dependent on acquiring one or more B-vitamins from diet and/or prototrophic microbes *via* committed salvage pathways. Here, we report the stability of gut microbiota using humanized gnotobiotic mice and *in vitro* anaerobic fecal culture in the context of extreme variations of dietary B-vitamin supply as revealed by phylotype-to-phenotype prediction from 16S rRNA profiling and metabolomic measurements. The observed nearly unaltered relative abundance of auxotrophic species in gut communities in the face of diet or media lacking B-vitamins or containing them in great excess (∼30-fold above normal) points to a strong contribution of metabolic cooperation (B-vitamin exchange and sharing) to the stability of gut bacterial populations.

## Introduction

Understanding metabolic interactions underlying assembly, maintenance, and dietary response of gut microbial communities is expected to provide a foundation for diagnostics, disease prevention, and therapeutic treatment of dysbiosis-related syndromes and diseases *via* rational nutritional supplementation. This concept is illustrated by studies evaluating the effects of complementary foods on healthy maturation of infant gut microbiota that were convincingly demonstrated in the context of severe acute malnutrition (Blanton et al., 2016). The human microbiota represents a complex assemblage of member species and a significantly larger set of gene functions. Collectively these gene functions provide the driving force for the establishment and maintenance of mutualistic relationships including both microbe-microbe and host-microbe interactions. One strategy microbes utilize to gain fitness advantage in the population is to engage in metabolic cooperation (Chassard and Bernalier-Donadille, 2006; Samuel and Gordon, 2006; Sonnenburg et al., 2006). The intensive selective pressure and fierce competition for nutrients in the gut, particularly in the colon, where bacterial populations are extremely dense (∼1 × 10^12^ bacteria/gram feces), may drive the subsequent shedding of genes involved in metabolic coding capacity thereby creating and/or deepening co-dependent relationships between groups of microbial species. It has been speculated that mutualism and synthrophic relationships abound in human microbiota. Evidence is accumulating to support this conjecture (Backhed et al., 2005; Mahowald et al., 2009; Fischbach and Sonnenburg, 2011). A combination of *in vitro* microbiological co-culture studies and the use of gnotobiotic mice have led to a broadened appreciation of the evolutionary strategies underlying functional interactions that define higher-order networks within microbial communities (Faith et al., 2010; Rey et al., 2010).

Metabolic interactions and the impact of dietary components on the microbiota have been studied in various ways. One of the best studied syntrophic networks operating in gut microbiota-host interactions pertain to processes involved in energy extraction from dietary glycans, sugar fermentation, and production of short chain fatty acids (Reichardt et al., 2018). When cultured with resistant starch 2 (RS2), *Ruminococcus bromii* generated significant quantities of reducing sugars but displayed a poor capacity for generalized carbohydrate utilization. However, when co-cultured with *Bacteroides thetaiotaomicron*, the total carbohydrate utilization of both species was increased (Ze et al., 2012). *Desulfovibrio piger* consumes ammonia, lactate, formate, and H_2_ generated by fermentation producing H_2_S. Fermentation efficiency is reduced by the accumulation of H_2_ that inhibits bacterial NADH dehydrogenases. Mutualism between *B. thetaiotaomicron* and *D. piger* has been reported (Rey et al., 2013). In co-colonized mice, *B. thetaiotaomicron* increases the fitness of *D. piger* by providing sulfate, compensating for the lack of sulfatase functions encoded by the *D. piger* genome. *D. piger* consumes products of fermentation H_2_ allowing fermentation efficiency of *B. thetaiotaomicron* to be maintained. The activities of some species alter the metabolic preferences of others. *Methanobrevibacter smithii* by an unknown mechanism directs *B. thetaiotaomicron* to preferentially ferment dietary fructans that generate acetate and formate that are subsequently consumed by *M. smithii* for methanogenesis (Samuel and Gordon, 2006). Mice co-colonization with *M. smithii* and *B. thetaiotaomicron* promoted increases in cell number of both species, indicating a mutualistic interaction.

In studies of dietary micronutrients, the impact of selenium was demonstrated using germ-free and gnotobiotic mice, revealing competition between the host and microbiota (Kasaikina et al., 2011). A selective effect of dietary vitamin A on fitness of distinct *Bacteroides* spp. was recently elucidated in gnotobiotic mice colonized by a defined consortium of human gut microbes (Wu et al., 2015). The metabolism of B-vitamins is of high significance since they are biosynthetic precursors of the universally essential cofactors such as TPP (derived from B1), FMN/FAD (from B2), NAD(P) (from B3), etc., drivers of numerous indispensable biochemical reactions in mammalian host and microbes alike. The mammalian host is strictly dependent on the dietary supply of all B-vitamins with the exception of niacin (B3), since in certain mammalian tissues NAD can be synthesized from tryptophan *via* by-passing nicotinate (-amide) requirement. Unlike other micronutrients mentioned, B-vitamins can be synthesized by many bacterial species. However, our *in silico* analysis of available reference genomes revealed that on average 20–30% of species in gut microbial communities (by relative abundance) lack the machinery to synthesize some of the essential B-vitamins (Rodionov et al., 2019). The large fraction of B-vitamin auxotrophs in human gut microbiota implies their strong dependence on the exogenous supply of these micronutrients from the diet and/or v*ia* syntrophic sharing between prototrophic and auxotrophic species.

A potentially important contribution of B-vitamin sharing in microbial communities is supported by several considerations: (i) a limited bioavailability of dietary B-vitamins for microbes in the distal gut due to their active absorption by the host; (ii) relatively low micronutrient requirements (as compared to essential amino acids or other building blocks) (iii) reported precedents of community-wide B-vitamin exchange illustrated by a prototroph-centered microbial consortium from Hot Lake (Romine et al., 2017), and a positive fitness impact provided by B12 prototrophs to a B12 auxotroph in gnotobiotic mice (Goodman et al., 2009).

Based on these considerations, we sought to evaluate the hypothesis that members of microbial communities engage in B-vitamin sharing as a potentially dominant form of mutualism. We hypothesized that a strong contribution of metabolic cooperation would manifest in relatively small or no changes in relative abundance of auxotrophic species even upon substantial changes in the supply of B-vitamins from growth media or diet. To address this hypothesis, we have applied our methodology of genomics-based predictive phenotype profiling, based on projection of 16S rRNA species enumeration of microbiome samples over a curated collection of ∼2,200 sequenced gut resident genomes (Rodionov et al., 2019) for the analysis of fecal samples from humanized gnotobiotic mice that were fed diets supplemented with varying B-vitamin levels (from complete deprivation to 30-fold excess over normal). To reduce the complexity of three-way (microbiota-diet-host) interactions, these studies were extended to *in vitro* anaerobic fecal cultivation and metabolomic measurements. We also used diagnostic *Escherichia coli* B3 and B5 auxotroph mutant strains to test the ability of a panel of prototrophic isolates from human microbiome to support their growth in the absence of respective vitamins in the growth media.

The obtained results, both *in vivo* and *in vitro*, provided strong support of our central hypothesis revealing a remarkable stability with respect to widely varying levels of exogenously supplied micronutrients (B-vitamins). We use the term stability in this context of the robustness of the auxotrophic component of the studied microbial communities. This observation implies that at least some of the prototrophic species operate as efficient *donors* of micronutrients (B-vitamins), to satisfy the growth requirements of a sizeable fraction of gut auxotrophs.

In addition to the fundamental and practical implications of these findings, the established integrative methodology will enable an extension of these studies toward microbial communities with varying auxotroph representation, under different types of diets or other physiological conditions of the mammalian host, e.g., health vs. disease.

## Materials and Methods

### *Escherichia coli* K-12 Growth Conditions

*Escherichia coli knockout* mutants. ΔNadA (B3 auxotroph), ΔPanC (B5 auxotroph), and ΔUxaC (as an isogenic proxy for wild-type with respect to B-vitamin synthesis) from KEIO collection (Baba et al., 2006) were grown at 37°C in M9 solid or liquid medium with or without vitamin B3/B5.

### Isolation of Gut Prototrophs

A pool of four human feces was plated on chemically defined medium without any B-vitamin under anaerobic conditions. 90 colonies were picked, grown in liquid culture and glycerol stocks were prepared. 85 out of 90 isolates grew well aerobically. Full-length 16S rRNA amplicons were subjected to Sanger sequencing followed by BLAST search against bacterial 16S rRNA database at NCBI for taxonomic identification of each isolate. All but four were *E. coli* strains.

### Rescue Experiments With Wild Type (WT) *E. coli*

Wild type *E. coli* K-12 was grown in M9 medium without any B-vitamin. Culture supernatant or conditioned medium was collected, filter sterilized and stored at -20°C. O.D._600_ of cultures were measured to normalize the volume of culture supernatant to be used for ΔNadA or ΔPanC rescue. To prepare recipient cells, ΔNadA or ΔPanC was grown overnight in M9 medium with 20 μM vitamin B3 or B5, respectively. The cells were collected by centrifugation, washed with vitamin free M9 medium and resuspended in M9 medium without vitamin B3 or B5, respectively. Cells were incubated at 37°C for 6 h to deplete endogenous pools of B-vitamin. Cells were diluted in fresh medium to an O.D._600_ < 0.001 and 100 μL of culture were mixed with 100 μL conditioned medium derived from fecal *E. coli* isolates in a 96 well plate and growth was monitored every 20 min.

### *In vitro* Anaerobic Cultivation

Anaerobic culture was performed in an anaerobic chamber containing 9% hydrogen and 91% nitrogen (Coy Laboratory Products, Grass Lake MI). A chemically defined medium (CDM) was used for all cultures. CDM contains 50 mM HEPES, 2.2 mM KH_2_PO_4_, 10 mM Na_2_HPO_4_, 60 mM NaHCO_3_, 4 mM of each amino acid, except leucine (15 mM), 10 mL ATCC, Trace Mineral Supplement. CDM contained nucleoside bases (100 mg/L), inosine, xanthine, adenine, guanine, cytosine, thymidine and uracil (400 mg/L). CDM contained choline (100 mg/L), ascorbic acid (500 mg/L), lipoic acid (2 mg/L), hemin (1.2 mg/L) and myo-inositol (400 mg/L). Resazurin (1 mg/L) was added to visually monitor dissolved oxygen. The pH of the media was adjusted to 7.4. Porcine gastric mucin (1% w/v) replaced glucose. Normal B-vitamin concentrations were as follows: B1 (6 mg/L); B2 (6 mg/L); B3 (30 mg/L); B5 (16 mg/L); B6 (7 mg/L); B7 (0.2 mg/L); B9 (2 mg/L); and B12 (2.5 mg/L). Excess B-vitamin concentrations were as follows: B1 (180 mg/L); B2 (180 mg/L); B3 (900 mg/L); B5 (480 mg/L); B6 (210 mg/L); B7 (6 mg/L); B9 (60 mg/L); and B12 (75 mg/L). Approximately 1 × 10^6^ pooled fecal bacteria were inoculated in pre-reduced CDM with and without B-vitamins (Table 1) and grown to approximate saturation for 3 days at 37°C.

**Table 1:** Experimental media and diets used *in vitro* and *in vivo.*

B-vitamins:		B1	B2	B3	B5	B7	B6	B9	B12
		Thiamine	Riboflavin	Niacin	Pantothenate	Biotin	Pyridoxine	Folate	Cobalamin
Respective cofactors:		TPP	FMN/FAD	NAD/NADP	Coenzyme A	Biotinyl-ACP	PLP/PMP	THF/DHF	(Ado)-Cob
**Diet (Media)**		**Vitamin supplementation (mg/L):**							
AD	All vitamins deficient	–	–	–	–	–	–	–	–
AN	All vitamins normal	6	6	30	16	0.2	7	2	2.5
AE	All vitamins in excess	180	180	900	480	6	210	60	75
4D4N	4 deficient/4 normal	–	–	30	–	–	7	2	2.5
4E4N	4 in excess/4 normal	180	180	30	480	6	7	2	2.5
7D1N	7 deficient/1 normal	–	–	–	–	–	–	–	2.5
7E1N	7 in excess/1 normal	180	180	900	480	6	210	60	2.5


### Humanized Gnotobiotic Mice and B-Vitamin Diets

The accuracy of B-vitamin biosynthesis phenotypic predictions is robust but highly dependent on the accuracy of species enumeration based on 16S rRNA profiles. The HMP reference genome sequencing efforts, while highly useful, are heavily biased to human isolates. We therefore elected to use humanized gnotobiotic mice to study the impact of B-vitamin dietary extremes. In order to establish gnotobiotic mice harboring gut microbes with high diversity and without bias that may be contributed by a single human donor, we conducted fecal gavage using five healthy human donor samples (IRB 2009019551EP, under written informed consent) sequentially at 1 week intervals. After the final gavage, mice were maintained for 4 weeks and fecal pellets from humanized gnotobiotic mice were collected. New germ free mice were gavaged twice (day 0 and day 2) with fecal pellets derived from humanized mice. Fecal pellets were collected on Day 7 (controls). B-vitamin supplementation in drinking water (Table 1) was maintained for 4 weeks and fecal pellets were collected for 16S rRNA sequencing and cecal contents were collected for LC/MS measurement of B-vitamins. Vitamin deficient and surplus diet studies were performed using a modified AIM-93M diet produced at Harlan Laboratories as has been published (Benight et al., 2011). This diet uses a vitamin free casein base, with cornstarch, maltodextrin sucrose, soybean oil and cellulose as the main source of calories, lipids, and fiber. All diets were vacuum-sealed and irradiated for sterility by the manufacturer prior to introduction into the gnotobiotic environment (Kasaikina et al., 2011). All mouse experiments were performed using protocols approved by Johns Hopkins University Animal care and Use Committee (IACUC number MO14M345).

### Bacterial DNA Extraction and 16S rRNA Sequencing

Fresh mouse stool samples were collected and frozen on dry ice and stored at -80°C. The bacterial DNA was extracted using the QIAmp Fast DNA Stool Mini Kit (Qiagen) with the additional step of using a Mini-Beadbeater-16 (Biospec Products) for 5 min to ensure uniform and efficient cell lysis. Library preparation was performed following Illumina’s protocols, targeting the V3–V4 region. 16S ribosomal DNA was amplified with PCR using forward:

5^′^-TCGTCGGCAGCGTCAGATGTGTATAAGAGACAGCCTACGGGNGGCWGCAG 3^′^, and reverse:

5^′^GTCTCGTGGGCTCGGAGATGTGTATAAGAGACAGGACTACHVGGGTATCTAATCC 3^′^.

Adapter and barcode sequences for dual indexing were also used as described in the 16S Metagenomic Sequencing Library Preparation protocol. PCR clean-up steps were done with QIAquick 96-PCR Clean-up kit (QIAgen), and library quantification was done using KAPA Library Quantification Kit for Illumina platforms (KAPA Biosystems). The Experion system (Bio-Rad) was used to analyze the DNA concentration and purity of pooled libraries. All 16S rRNA sequence reads from *in vivo* and *in vitro* analyses have been deposited at NCBI under the Bioproject accession numbers PRJNA545546 and PRJNA545535, respectively.

### Predictive B-Vitamin Phenotype Profiling

Genomics-based phenotype profiling was performed using our *phylotype-to-phenotype prediction pipeline*, which allows us to translate the 16S rRNA-based phylogenetic profiles (list of phylotypes and their relative abundance) of gut microbiome samples into respective *Community Phenotype Profiles* providing tentative fractional representation of B-vitamin auxotrophy/prototrophy phenotypes in these samples. These predictions are based on genomics-based metabolic reconstruction of respective subsystems (pathways) in RAST/SEED genomic platform (Overbeek et al., 2014) as described in details (Rodionov et al., 2019). Briefly, all identified phylotypes were mapped onto a reference collection of RAST-annotated genomes representing the human microbiome. The reference collection of 2,228 genomes representing the human gut microbiota was compiled based on (i) genomes collected by the MetaHIT consortium (Qin et al., 2010); (ii) genomes of bacteria isolated from the human gastrointestinal tract collected by the human microbiome reference genome database (Human Microbiome Project Consortium, 2012); (iii) collection of ∼1,000 cultured species of the human gastrointestinal microbiota (Rajilic-Stojanovic and De Vos, 2014), and by their mapping into the PATRIC genomic database (Antonopoulos et al., 2017) and further adding of ∼250 additional genomes of their closely related species.

*In silico* metabolic reconstructions in SEED subsystems were based on functional gene annotation using homology-based methods and three genome context techniques: (i) clustering of genes on the chromosome (operons), (ii) co-regulation of genes by a common regulator or a riboswitch, and (iii) co-occurrence of genes in a set of related genomes. These context-based techniques were used to disambiguate paralogs with related but distinct functions (most notably transporters) and fill-in gaps (“missing genes”) in the inferred biochemical pathways. The subsystems-based approach to reconstruction B-vitamin metabolism (Osterman et al., 2010) and its extension to human gut microbial species (Magnusdottir et al., 2015) were previously described.

Based on metabolic pathway topology and the genomic distribution of vitamin metabolism enzymes and transporters, we established phenotype rules describing various pathway variants for each vitamin/cofactor. Binary phenotype values (“1” for prototrophy and “0” for auxotrophy) for each out of eight analyzed B-vitamins and each of individual genomes were obtained via translation of the assigned vitamin pathway variants and combined together into a binary phenotype matrix (BPM).

We used a development version of *Phenobiome Phenotype Profiler* tool (courtesy of PhenoBiome Inc., Walnut Creek, CA, United States) ^1^ to map phylotypes to the reference collection of genomes based on their taxonomic assignments and calculated weights (*w*) at three taxonomic levels as described in more details in Rodionov et al. (2019). Briefly, this procedure includes the following steps: (i) mapping of all identified phylotypes to our reference collection of 2,228 curated genomes representing human gut microbiome; (ii) for each mapped phylotype, a phenotype value assignment (on the scale 0–1) is performed by averaging of binary phenotypes (0 or 1) for individual genomes comprising a phylogenetic neighborhood of each phylotype; (iii) multiplying a deduced phenotype value of each detected phylotype by its relative abundance (%); (iv) taking a total of obtained individual values for each replicate; and (v) averaging the obtained total amounts for all replicates within the group and computing respective STDEV (errors). For phylotypes precisely mapped at the species level, equal weights are assigned to genomes of all strains in the collection that belong to same species (for the purpose of averaging and probabilistic phenotype assignment). For phylotypes that could not be mapped at species level, phenotype averaging is performed at genus level applying with equal weights assigned to all species within a genus. A similar approach to averaging/weighting is applied to phylotypes mapped only at family level. Phylotypes that do not map at the family level (typically ≤0.5% by abundance) were excluded from phenotype prediction.

The mapped phylotypes and the reference BPM were further used to calculate Community Phenotype Matrix (CPM) capturing all individual binary phenotype values (*p*) multiplied by weights (*w*). Each value in CPM reflects a relative contribution of each phylotype to the analyzed phenotype (Supplementary Tables S1B, S2B). Each Community Phenotype Index (CPI), a probabilistic estimate of a fraction (%) of prototrophic cells in a given community that have a functional pathway for the *de novo* biosynthesis of a given B-vitamin, was calculated as total of respective CPM values (contributions) multiplied by relative abundances (*A*) of all individual phylotypes in a given sample:

$$\mathrm{CPI}=\sum_{i}A_{i}\sum_{m}w_{i,\;m}p_{m}$$

To account for variations in 16S rRNA gene copy number in different species, renormalization of OTU count data was optionally performed using pan-taxa statistics for the ribosomal RNA operon copy number provided by the rrnDB database (Stoddard et al., 2015). Each OTU’s relative abundance was divided by the simple mean of all known 16S rRNA gene copy numbers for a particular species, genus or family based on the best match between the OTU’s assigned taxonomy and the corresponding rrnDB taxonomy. At the end all relative abundances for each sample were rescaled to sum up to one.

### LC/MS Measurements of Selected B-Vitamins in *in vitro* Cultures and Cecal Samples

Human fecal pools were used to inoculate chemically defined medium with B-vitamins (conditions as indicated in Table 1). The cultures were grown anaerobically for 48 h. O.D._600_ was determined. Cells were collected by centrifugation and supernatants were stored separately at -80°C until further processing. At the time of sacrifice, cecal contents were collected and immediately frozen. The samples were stored at -80°C until further processing.

## Results

### Community-Wide B-Vitamin Sharing *in vivo*: Humanized Gnotobiotic Mouse Model

**FIGURE 1:**
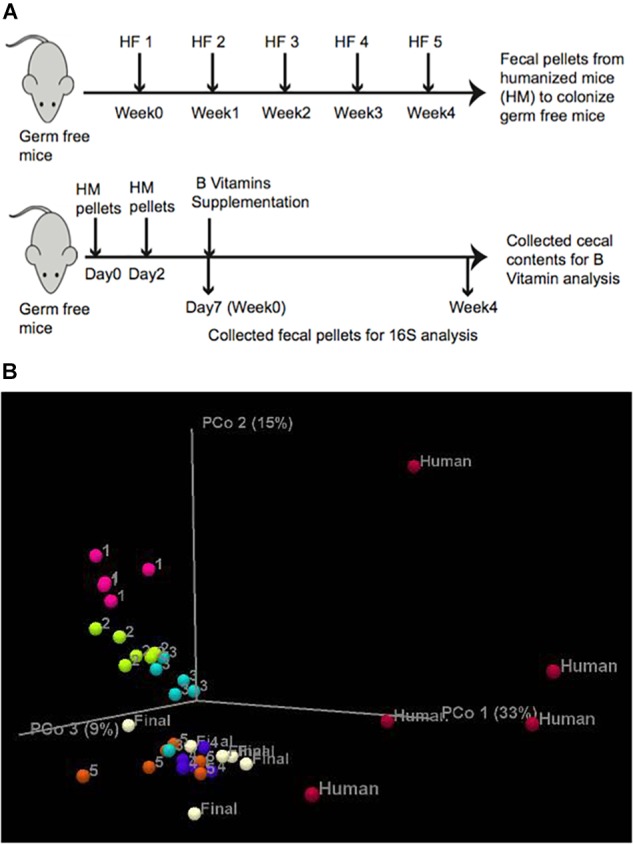
*In vivo* experimental design. **(A)** Humanization of germ free mice was carried out by successive gavage with human feces derived from 5 healthy human subjects (HF) at 1 week intervals. After the final gavage, mice were maintained for 4 weeks and fecal pellets from humanized mice (HM) were collected. Lower panel. Germ free mice were gavaged twice with fecal pellets derived from humanized mice at an interval of 2 days. Fecal pellets were collected on Day 7 (controls). B-vitamin supplementation in drinking water (Table 1) was maintained for 4 weeks and fecal pellets were collected for 16S rRNA sequencing and cecal contents were collected for LC/MS measurement of B-vitamins. **(B)** PCoA analysis of mouse humanization. Unweighted Uni-Frac β-diversity of communities at different stages of humanization.

To test whether the abundance of B-vitamin auxotrophs in gut microbial communities are affected by the dietary supply of these micronutrients, we have established a model using gnotobiotic mice colonized with human fecal samples. In order to increase the diversity of gut communities we sequentially introduced fecal communities one subject at a time. Fecal slurries from subject 1 was introduced into C57BL/6 germ free (GF) mice (*n* = 5) by oral gavage. After 1 week, fecal samples from subject two were introduced and so on for a total of five donors (Figure 1A and Supplementary Table S1F). The composition and β-diversity of communities following the first through third fecal gavage cleanly separated in PCoA plots. Subsequent gavage had no discernable impact in altering community composition (Figure 1B). We did not observe significant differences in microbiota diversity using sequential colonization, although the source of phylotypes (strains) in the final mice were derived from distinct donors (not shown).

To achieve greater microbiota uniformity at baseline, 1 week after the final oral gavage, feces were collected and pooled in equal mass and used to colonize new GF mice (Figure 1A). The resulting humanized gnotobiotic mice (*n* = 5/group) were provided a custom chow devoid of B-vitamins. B-vitamins were supplied in the drinking water in varying quantities to assess the impact of extremes in B-vitamin availability on phylogenetic and predicted phenotype profiles of gut microbial communities. Seven formulations were tested representing vitamin *D*eficiency, *N*ormal or *E*xcessive supply of *A*ll 8 B-vitamins (“AD”, “AN,” and AE”, respectively) along with four intermediate formulations containing various levels of different subsets of these vitamins (Table 1). Normal B-vitamin concentrations were defined as that present in a standard diet. Mice were maintained on these diets for 4 weeks. 16S rRNA sequence analysis was performed on fecal samples collected at 0 and 4 weeks after initiating B-vitamin varied diets. The analysis of varied B-vitamin diets required the use of a custom chow that altered the gut microbiota (not shown), therefore, we focused on end-point analysis of microbial communities after 4 weeks of specified B vitamin diets.

### Phylogenetic Profiling of Microbial Communities

We sequenced the V3–V4 region of 16S rRNA present in fecal communities. In order to obtain the highest precision enumeration of observed taxa, we used phylotype-based, rather than OTU-based analyses to avoid over-binning of distinct bacterial species (see Methods). This approach allowed us to map over 90% of the relative total abundance of taxa to named species with >98% sequence identity. Approximately 8% of taxa displayed sequence identity to named taxa of 95–98%, approximating genus level assignments and less than 2% of enumerated taxa could only be assigned at the family-level (Supplementary Table S1A). The high precision of species enumeration facilitated high confidence B-vitamin biosynthetic phenotype predictions using available reference genome sequence assemblies. We observed inter-mouse variation in the composition of fecal microbiota at baseline. The relative abundance of Akkermansia genus (∼ 27%), driven almost exclusively by a single taxon with high similarity to *Akkermansia muciniphila*, is significantly higher than typically observed in human microbiome samples (Supplementary Figure S2). Similarly, taxa with identity to *Muribaculum intestinale* colonized the mouse efficiently and established relative abundance higher than that observed in HMP data sets. With those exceptions, the humanized mouse model is reasonably representative of taxa and proportionality observed in human microbiota. A longitudinal comparison of phylogenetic profiles in fecal samples at baseline and at the end of 4 week on B-vitamin varied diet revealed no trends above mouse-to-mouse variations attributable to dietary effects (Supplementary Tables S1A,B).

### Predictive Phenotype Profiling of Microbial Communities

All bacterial phylotypes enumerated by 16S rRNA analysis were assigned predicted auxotrophy/prototrophy phenotypes for eight B-vitamins (see Section Materials and Methods). Results of these predictions are illustrated for the 20 phylotypes displaying the highest average relative abundance (Table 2), and for the complete set of 207 mapped phylotypes (Supplementary Table S1C). Predicted B-vitamin prototrophy phenotypes are captured in a format of Binary Phenotype Matrix (BPM) where the probability of *de novo* synthesis of each B-vitamin-related essential cofactor is deduced from genomics-based reconstruction of respective biosynthesis/salvage pathways (Rodionov et al., 2019). The species for which phenotype predictions were available was compared to profiled species. Matching at the species (S) level achieved ∼90% coverage by relative abundance, yielding precise phenotype predictions with deduced probability values, 1 = prototrophy or 0 = auxotrophy. In relatively rare cases, phenotype microheterogeneity at the strain level was noted. This probability (phenotype index) is approximated by the average values for all available reference genomes. Thus, for *Akkermansia muciniphila*, only one out of four reference genomes has a complete pathway of B12 *de novo* synthesis (prototrophy), while the other three strains lack this pathway and rely on salvage of exogenous B12 (auxotrophy), therefore, a B12 prototrophy index is estimated by a value 0.25 (Table 2).

**Table 2:** Binary Phenotype Matrix of predicted B-vitamin phenotypes (prototophy) for most abundant 20 species representing microbial community established in gnotobiotic mouse model with humanized gut microbiome.

Mapping to reference collection			Predicted B-vitamin prototrophy^3^								Abund.^4^
		
Matching species	Mapping level^1^	Genomes^2^	B1	B2	B3	B5	B6	B7	B9	B12	%
*Akkermansia muciniphila*	**S**	4	1	1	1	1	1	1	1	0.25	26.3%
*Muribaculum intestinale*	**S**	1	1	1	1	1	1	1	1	0	10.2%
*Faecalibacterium prausnitzii*	**S**	6	0	0.67	0	0	1	0	0	1	9.8%
*Bacteroides xylanisolvens*	**S**	4	1	1	1	1	1	1	1	0.25	4.5%
*Blautia glucerasea*	**S**	1	1	0	1	0	1	0	1	1	3.6%
*Parabacteroides distasonis*	**S**	4	1	1	1	1	1	1	1	1	3.1%
*Murimonas intestini*	**F**	224	0.48	0.56	0.7	0.25	1	0.15	0.49	0.81	3.0%
*Phascolarctobacterium faecium*	**G**	1	1	1	0	1	1	1	1	1	2.5%
*Bacteroides fragilis*	**S**	6	1	1	1	1	1	1	1	1	2.3%
*Bacteroides uniformis*	**S**	7	1	1	1	1	1	1	1	1	2.2%
*Bacteroides vulgatus*	**S**	6	1	1	1	1	1	1	1	1	2.0%
*Coprococcus eutactus*	**S**	1	1	1	1	1	1	0	1	0	1.6%
*Sutterella massiliensis*	**G**	4	0	1	0	0	1	0.5	1	0	1.6%
*[Eubacterium] rectale*	**S**	3	1	1	1	1	1	0	0	1	1.4%
*Bacteroides stercoris*	**S**	2	1	1	1	1	1	1	1	1	1.2%
*Blautia faecis*	**G**	23	0.75	0.31	0.77	0.07	1	0.19	0.86	1	1.1%
*Bacteroides stercorirosoris*	**S**	1	1	1	1	1	1	1	1	0	0.9%
*Bacteroides cellulosilyticus*	**S**	3	1	1	1	1	1	1	1	1	0.8%
*Ruminococcus champanellensis*	**S**	2	0	0	1	0	1	0	1	0	0.8%
*Parabacteroides goldsteinii*	**S**	3	1	1	1	1	1	1	1	1	0.8%


*^1^“Mapping level”: indicates whether a given phylotype (represented by species names in the first column) could be matched to our Reference Collection of genomes at the level of individual species (“S”) or only at the level of a corresponding genus (“G”) or family (“F”). ^*2*^“Genomes”: the number of genomes available in Reference Collection corresponding to the indicated mapping level of a given phylotype that were used for B-vitamin phenotype prediction (as explained in the next comment). ^*3*^“B-vitamin prototrophy (Community Phenotype Index, CPI)”: CPI values reflect a likelihood (probability on a scale from 0 to 1) of a given phylotype to be a prototroph for a respective B-vitamin (B1 thru B12). For phylotypes mapped at the level of species (S in column B), CPI value is estimated by averaging respective values (“1” for protortophy and “0” for auxotrophy) predicted from metabolic reconstruction of B-vitamin pathways in individual genomes of all strains/isolates of this species that are presently captured in Reference Collection. For phylotypes mapped at the level of genus, CPI values are first computed for every species within this genus (as described above) and the mean of the obtained values is assigned as a G-level CPI. A similar two-step procedure (species-level averaging) is applied to phylotypes mapped at family level. A complete set of predictions for all mapped phylotypes are provided in Supplementary Table S1C). ^*4*^“Abund.”: relative abundance averaged for all fecal samples obtained before the start Variable B-vitamin feeding arm of the study (Week 0), see Supplementary Table S1A. Gray-white values are in the range from 0 to 1.*

Predictive precision is generally lower for species that are not present in the current Reference Collection, necessitating matching at the genus level (G), representing ∼8.5% by relative abundance of the community. Notably, many B-vitamin phenotypes are rather conserved even at the genus level yielding predominantly binary (1 or 0) values as in *Bacteroides, Parabacteroides*, whereas *Peptoniphilus* spp., display significant strain level heterogeneity (Table 2). Such heterogeneity limits the predictive precision. Only ∼2% by relative abundance were mapped at low resolution to the family (F) level. No phenotype assignments were made for phylotypes mapping at higher taxonomic levels.

Notably, B12 mono-auxotrophs (12 species) and omni-prototrophs (14 species) account for the largest fraction of the analyzed microbiota with a combined average relative abundance of 47–50% and 16–19%, respectively (Supplementary Table S1C). Indeed, the two most abundant species, *Akkermansia muciniphila* (26%) and *Muribaculum intestinale* (10%) are B12 mono-auxotrophs (Table 2). Nevertheless, a number of multi-auxotrophs, most notably *Faecalibacterium prausnitzii* (average abundance ∼9.8%), are maintained in high abundance. A total of 28 species auxotrophic for at least 3 (and up to 6) distinct B-vitamins comprise ∼20% of the population by relative abundance (Supplementary Table S1B). The observation that auxotrophy for vitamin B12 is generally higher as compared to other B-vitamins may be explained by the fact that B12 is the only representative of B-vitamins, which is truly dispensable in many microbial species. Indeed, the main (although not the only) essential metabolic role of B12 is in the biosynthesis of methionine as cofactor of cobalamin-dependent methionine synthase MetH. However, many microbial species contain an alternative cobalamin-independent methionine synthase MetE. Greater than 60% of methionine prototrophs (among 1,750 genomes) in our mcSEED reference collection, encode only MetE (680 genomes) or both MetH and MetE (460 genomes). The latter group of organisms may still improve fitness in the presence of exogenous B12 (MetH is known to be more catalytically processive than MetE), but this requirement is much less stringent than any other B-vitamin auxotrophy. Therefore, it is not surprising that this pathway, one of the longest and most costly among B-vitamin related co-factors, is readily lost in many MetE-driven species. A completely different situation exists in many *Bacteroides* spp. and some other prominent groups of gut microbial species that possess only MetH but are at the same time B12 auxotrophs that are fully dependent on its exogenous supply (Goodman et al., 2009).

These observations led us to pose two important questions. Does the ability to carry out *de novo* synthesis of all essential co-factors dictate their abundance relative to multi-auxotrophs? Second, to what extent is the fitness of multi-auxotrophs impacted by the dietary supply of B-vitamins compared to possible B-vitamin sharing provided by prototrophic donors? To address these questions, we first performed a global comparison of Community Phenotype Signatures (CPS_ of B-vitamin auxotrophy computed for all samples obtained in B-vitamin varied diets studies. Each of the eight values (one for each vitamin) comprising CPS is calculated as a community-wide total of contributions to auxotrophy (%) by each individual phylotype. These contributions are computed by multiplication of inverse phenotype values (1 – Prototrophy Index) from Supplementary Table S2B over their respective relative abundances. The resulting CPS values are averaged within each dietary group (Supplementary Table S1D).

**FIGURE 2:**
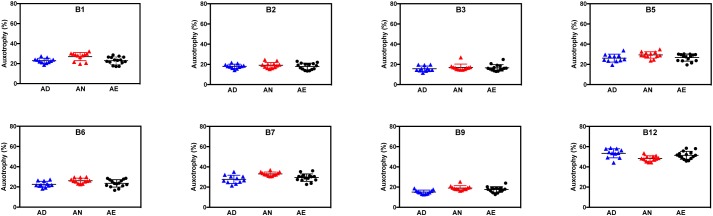
Representation of B-vitamin auxotrophs in microbial communities from humanized gnotobiotic mice fed diets supplemented with different levels of B-vitamins. Community-wide phenotype signatures in the form of auxotrophy representation (%) for each of the eight B-vitamins (as indicated), which were deduced from 16S rRNA profiling of fecal samples (based on data in Supplementary Tables S1A,B), are plotted for all individual animals from three groups provided one of the three diets for 4 weeks; (*AD*) a vitamin-free chow with no B-vitamins in the drinking water or the same chow but B-vitamins *normal* (*AN*) or *excessive* (*AE*) were supplemented in drinking water (Table 1).

For nearly all B-vitamins the observed auxotrophy representation was in the 20–30% range, with a notable exception of B12 auxotrophy level (∼50%), which is consistent with a trend discussed above. Most importantly, no vitamin-specific dependencies (beyond intragroup variations) could be detected between different diets lacking B-vitamins as well as containing them in normal or excess quantities as illustrated for AD, AN, and AE dietary groups (Figure 2). PCoA using Bray-Curtis β-diversity of communities after 4 weeks of AD, AN, and AE did display separation (Supplementary Figure S1A), however, the observed differences did not show any diet-dependent trends and did not impact the frequency of B-vitamin auxotrophs.

To assess whether the observed stability manifests only at the community level as a result of gross (community-wide) averaging, we compared the relative abundance of numerous individual multi-auxotrophic species present in extreme dietary conditions (Supplementary Table S1B). To improve statistics, we aggregated the results obtained for three vitamin-deficient diets (AD, 4D4N, and 7D1N), which have a common subset of deficient vitamins B1, B2, B5, and B7. These were compared to aggregated results from three other diets (AE, 4E4N, and 7E1N), where the same subset of B-vitamins is present in excessive amounts. Notably, of 26 reliably detectable species (≥0.01%) with unambiguous auxotrophy for at least 3 out of 4 B-vitamins (B1, B2, B5, and B7), only five showed a modest (average 0.2–0.8%) but statistically significant increase (P-value < 0.04) in the presence of excessive supply of respective vitamins (Supplementary Table S1B). The most consistent increase in relative abundance (∼2-fold) was observed for two B1/B2/B5/B7 auxotrophs, *Romboutsia timonensis* and *Negativibacillus massiliensis*, and one B1/B2/B5 auxotroph [*Clostridium*] *saccharolyticum* (Supplementary Figure S3). Most other multi-auxotrophs, including the most prominent *Faecalibacterium prausnitzii*, showed only minor and statistically insignificant differences in abundance between the two extreme dietary series (Supplementary Table S1B).

Overall, both types of data analysis (community-wide and species-by-species) described above yield consistent results and suggest tentative answers to both questions formulated above. Since the excessive supply of all B-vitamins (diet AE) fails to increase the overall representation of any B-vitamin auxotrophs as compared to both AN and AD diets (Figure 2), none of these vitamins appears to be a limiting micronutrient for respective auxotrophs. Therefore, it is unlikely that the observed overall higher representation of prototrophs is defined solely by their independence from the supply of exogenous B-vitamins but rather driven by other fitness determinants. Additionally, the observation that B-vitamin-deficient diet did not drive decreases in the overall representation of auxotrophs suggests that they are not dependent on dietary vitamin supply and capable of satisfying their micronutrient requirements by the salvage of B-vitamins released by prototrophic members of the community.

### LC/MS Measurements of Selected B-Vitamins in Unfractionated Cecal Samples

**FIGURE 3:**
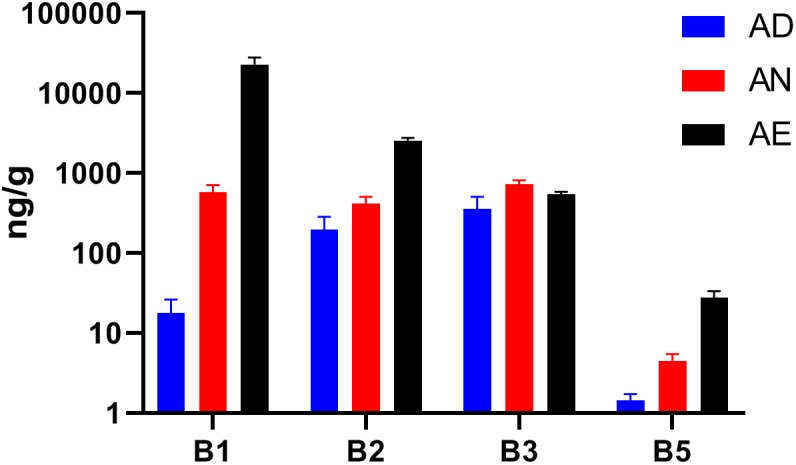
Levels of selected B-vitamins in cecal samples from humanized gnotobiotic mice fed diets supplemented with varying B-vitamins. The levels of four vitamins (B1, B2, B3, and B5) were determined by LC–MS in the cecal samples from mice fed the same three diets (AD, AN, and AE) as in Figure 2 (using 3 replicates for each of the three diets) and plotted as averaged values (in ng/g cecal material) with *SD* as error bars.

Our ability to interpret the diet-dependent phylogenetic and phenotype profiling results is contingent on the assumption that extreme changes in dietary B-vitamin supply (excess or deficiency) may indeed affect the availability of these micronutrients in the distal gut. Given the unknown efficiency of intestinal absorption, which may differ for different vitamins, we have tested this assumption by LC/MS measurements of four reliably detected B-vitamins (B1, B2, B3, and B5) in cecal samples collected from all seven groups of mice (Supplementary Table S1E). With the exception of niacin (B3), the measured levels of B-vitamins in all samples displayed changes that were in qualitative agreement with their dietary supply (Figure 3). The largest fold-change between all three diets was observed for vitamins B1 and B5. The highest basal levels (in AD diet) was observed for vitamins B2 and B3, likely reflecting the higher net production of these vitamins by the microbial community. This effect may be masked by a particularly active absorption by the host combined with robust biogenesis (and sharing) by prototrophs, e.g., vitamin B3. Furthermore, the measured B-vitamin levels do not distinguish intracellular and extracellular pools of cecal material. Despite these limitations, the observed trends generally confirm that B-vitamin levels can be manipulated in the distal gut, thus supporting that microbiota-wide vitamin sharing contributes to community stability in the face of varying dietary supply of these micronutrients.

### Community-Wide B-Vitamin Sharing *in vitro*: Anaerobic Fecal Culture Model

We used *in vitro* anaerobic culturing to further test the vitamin sharing hypothesis in a simplified model system, which allows us to eliminate uncertainties related to vitamin absorption or sharing by the host. We performed a series of *in vitro* anaerobic cultivation studies of fecal pools, derived from four human subjects used for mouse humanization. We employed a chemically defined media (CDM) supplemented with porcine gastric mucin to enhance bacterial diversity. We generated seven media compositions representing deficiency, normal or excessive supply of different vitamin subsets (Table 1), to mirror the *in vivo* studies. Approximately 1 × 10^6^ fecal bacteria were inoculated in pre-reduced (CDM + mucin) media. For each media composition, the community outgrowth was conducted in replicate (*n* = 6).

### Phylogenetic Profiling

We sequenced the V3–V4 region of 16S rRNA present in fecal culture collected after 48 h of anaerobic growth, which led to ∼10 doublings. Data analysis was performed as for *in vivo* samples and yielded similar precision in mapping. More than 90% of the total relative abundance displayed >98% identity to a named species. The phylogenetic distribution of *in vitro* cultures was distinct from those observed *in vivo*. A comparison of the two datasets (Supplementary Figure S2) shows that only 5 out of the most abundant 20 genera are common to both. PCoA of resulting communities in AD, AN, and AE cultures failed to display any diet-dependent separation that achieved statistical significance between groups (Supplementary Figure S1B). A rank order of these shared families is also quite different, with the most notable decrease in representation of *Akkermansia, Muribaculum, Flavonifractor* and *Escherichia* show the most substantial expansion in the *in vitro* subcultures. We noted that variation between replicate cultures (*n* = 6) was greater than that observed *in vivo.* Despite this, no statistically significant media-dependent differences or even trends in phylogenetic profiles could be detected over the entire range of B-vitamin levels in the growth media (Supplementary Table S2A).

### Predictive Phenotype Profiling

**FIGURE 4:**
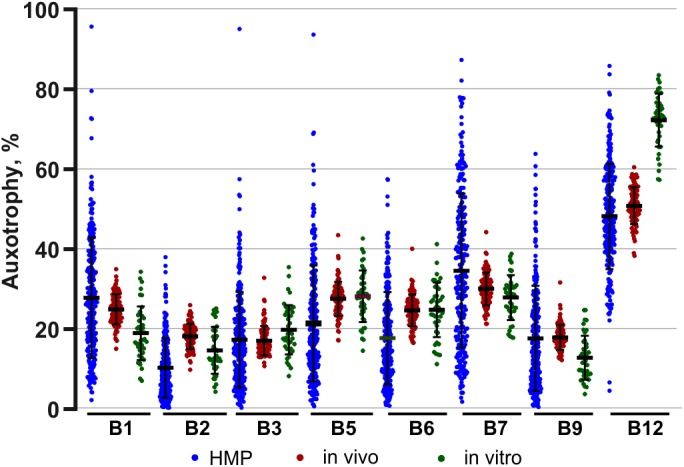
Comparison of B-vitamin auxotrophy phenotype signatures of *in vivo* and *in vitro* microbial communities and samples from human microbiome project (HMP). Phenotype signatures computed for samples obtained in all tested diets/media are shown by dot plots next to the aggregated signatures computed by the same approach for a subset of 313 fecal HMP samples.

The assignment of auxotrophy/prototrophy phenotypes for eight B-vitamins over the entire set of enumerated species (Supplementary Tables S2B,C) and computing community-wide phenotype signatures (Supplementary Table S2D) were performed as described for *in vivo* samples. Among the 95 taxa, accounting for 99.8% of reads, 81 (∼95%) were mapped at the level of species and 11 (∼4.5%) at the level of genus, leaving only a minor fraction unmapped or mapped at the family level (∼0.2% each). Notably, despite substantially different phylogenetic profiles of *in vitro* communities, B-vitamin phenotype signatures showed many similar trends, compared to those observed *in vivo*. Thus, the largest fraction of *in vitro* communities was represented by B12 mono-auxotrophs (10 species; ∼60% by relative abundance) (Supplementary Table S2C). Likewise, multi-auxotrophs (26 species, 3–8 B-vitamins) accounted for ∼26–27% of microbial communities. As in case of B12 mono-auxotrophs, the most abundant taxa in this category (Table 3) were distinct from those observed *in vivo*. Thus, *Flavonifractor plautii, Peptoniphilus grossensis* and *Finegoldia magna* are among the most prominent multi-auxotrophs *in vitro*, but not *in vivo* (compare with Table 2), while *Faecalibacterium prausnitzii*, the top multi-auxotroph *in vivo*, is below the detection level *in vitro*. The unique communities formed *in vitro* provided an independent means of comparing trends in B-vitamin prototrophy/auxotrophy representation to better distinguish host-dependent vs. microbial-dependent effects. Despite notable phylogenetic differences, the phenotype signatures of both *in vivo* and *in vitro* microbial communities are remarkably similar for nearly all B-vitamins except B12 (Supplementary Figure S4). Aggregated data on auxotrophy representation spanning all diets and time points, vary in a much narrower range as compared to microbial gut samples from the human microbiome project (HMP) dataset (Figure 4).

**Table 3:** Binary Phenotype Matrix of predicted B-vitamin phenotypes (prototophy) for most abundant 20 species representing microbial community established in anaerobic subculturing model of human gut microbiome.

Mapping to reference collection			Predicted B-vitamin prototrophy^3^								Abund.^4^
		
Matching species	Mapping level^1^	Genomes^2^	B1	B2	B3	B5	B6	B7	B9	B12	%
*Bacteroides thetaiotaomicron*	**S**	3	1	1	1	1	1	1	1	0	25.5%
*Escherichia fergusonii*	**S**	5	1	1	1	1	1	1	1	0	21.0%
*Flavonifractor plautii*	**S**	3	0	0	0	0	0	0	0	1	6.9%
*Peptoniphilus grossensis*	**S**	2	0	1	0.5	0	0	0	1	0	6.7%
*Finegoldia magna*	**S**	6	1	0	0	0	1	0	1	0	5.0%
*Bacteroides faecis*	**S**	2	1	1	1	1	1	1	1	0	4.5%
*Bacteroides vulgatus*	**S**	6	1	1	1	1	1	1	1	1	3.1%
*Bacteroides caccae*	**S**	3	1	1	1	1	1	1	1	0	3.1%
*Clostridium perfringens*	**S**	4	1	1	1	0	0	0	1	1	2.5%
*Phascolarctobacterium faecium*	**G**	1	1	1	0	1	1	1	1	1	1.8%
*Bacteroides massiliensis*	**S**	1	1	1	1	1	1	1	1	0	1.7%
*Bacteroides uniformis*	**S**	7	1	1	1	1	1	1	1	1	1.7%
*Agathobaculum desmolans*	**S**	1	0	0	0	0	0	0	0	1	1.2%
*Akkermansia muciniphila*	**S**	4	1	1	1	1	1	1	1	0.25	1.2%
*Pseudoflavonifractor phocaeensis*	**G**	3	0	0.5	0	0	0	0	0	0.5	1.2%
*Bacteroides ovatus*	**S**	7	1	1	1	1	1	1	1	0	1.0%
*Parabacteroides merdae*	**S**	4	1	1	1	1	1	1	1	1	0.9%
*Eggerthella lenta*	**S**	3	1	0	0	0.67	1	0	1	0	0.6%
*Flintibacter butyricus*	**S**	3	0	0	0	0	0	0	0	1	0.2%
*Pseudoflavonifractor capillosus*	**S**	1	0	0	0	0	0	0	0	1	0.2%


*^1^“Mapping level”: see comments to Table 2. ^*2*^“Genomes”: see comments to Table 2. ^*3*^“B-vitamin prototrophy (Community Phenotype Index, CPI)”: see comments to Table 2. A complete set of predictions for all mapped phylotypes are provided in Supplementary Table S2B). ^*4*^“Ave. abundance”: relative abundance averaged for samples obtained in media with normal levels of all B-vitamins (AN, *n* = 6), see Supplementary Table S2A. Gray-white values are in the range from 0 to 1.*

**FIGURE 5:**
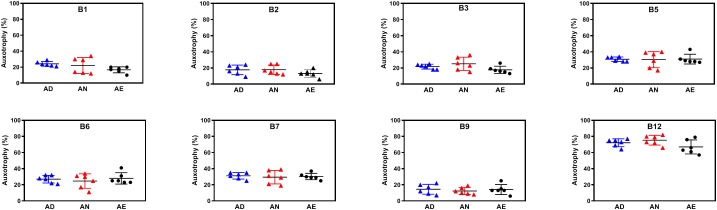
Representation of B-vitamin auxotrophs in microbial communities in anaerobic subcultures grown with different levels of B-vitamins. Community-wide phenotype signatures in the form of auxotrophy representation (%) *for* each of the eight B-vitamins (as indicated), which were deduced from 16S rRNA profiling of microbial cultures grown in defined media containing no added B-vitamins (*AD* – all deficient) and the same media supplemented with *normal* (*AN*) or *excessive* (*AE*) dietary levels of all eight B-vitamins (Table 1), are plotted for each replicate (*n* = 6).

Variations of media B-vitamin provisions in the *in vitro* cultures did not cause any significant changes at the level of community phenotype signatures in AD, AN, and AE media (Figure 5). Auxotrophy representation remained at the level of 20–30% for most B-vitamins and 10–15% for B9 (Supplementary Table S2D). The fraction of B12 auxotrophs in the community is substantially higher (65–75%), including mono- and multi-auxotrophs. We compared individual multi-auxotrophs in the cultures (AD, 4D4N, 7D1N) and (AE, 4E4N, and 7E1N). The growth rates of cultures in all media were comparable and no discernable differences in O.D._600_ were evident. None of these taxa displayed significant changes in relative abundance between extremes in B-vitamin provision in culture media (Supplementary Table S2C). We conclude that the observed higher representation of B12 mono-auxotrophs and prototrophs over multi-auxotrophs is not defined solely by the difference in their B-vitamin requirements, since a drastic increase of these (apparently non-limiting) micronutrients in the AE growth media as compared to AN media does not provide any detectable fitness advantages to the latter subset of species. B-vitamin requirements of multi-auxotrophic species are fully satisfied by vitamins released (excreted) by their prototrophic neighbors, as the observed auxotrophy representation is not decreased in B-vitamin-deficient AD media (Figure 5). These observations corroborated by direct metabolic measurements (see below) provided additional evidence in support of our central hypothesis that prototrophic microbes actively share B-vitamins enabling the sustained fitness of B-vitamin auxotrophs.

One of the important limitations of 16S rRNA-based community profiling is a well-appreciated non-equivalence of the measured relative abundance of reads and relative abundance of respective phylotypes. This is primarily due to substantial variations in 16S rRNA copy number between different species (and even strains). Indeed, renormalization on species-specific copy number data (see Section Materials and Methods) has an appreciable effect on phylogenetic profiles and respective phenotype profiles, both *in vivo* and *in vitro* (Supplementary Figure S5 and Supplementary Table S3). Notably, this renormalization did not reveal any new trends in the diet-dependent representation of auxotrophs in either dataset. For consistency, the illustrations in this article are shown without renormalization, which by itself, is not a perfect procedure due to incomplete and imprecise nature of 16S rRNA operon multiplicity data.

### LC/MS Measurements of Selected B-Vitamins

**FIGURE 6:**
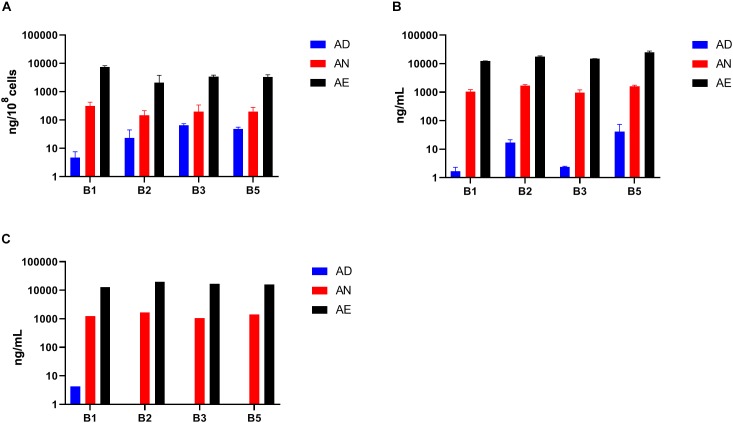
Levels of selected B-vitamins measured by LC-MS in samples of anaerobic subcultures grown with different levels of B-vitamins. **(A)** The levels of four vitamins (B1, B2, B3, and B5) in cell pellet samples (using three replicates for each of the three diets) from *in vitro* subcultures grown in the same three media (AD, AN, AE) as in Figure 5) and plotted as averages (ng/10^8^ cells) with *SD* as error bars. **(B)** B-vitamin measurements of respective supernatants (ng/mL). **(C)** B-vitamin measurements of respective media prior to inoculation (ng/mL).

To leverage the key advantage of host independent effects of B-vitamin sharing in *in vitro* communities we measured the quantities of the same four B-vitamins (B1, B2, B3, and B5) in spent culture supernatants and cell pellets from samples grown in three media AD, AN, and AE (Supplementary Table S2E and Figure 6). A comparison of fresh AD media (Figure 6A) and supernatants from samples grown in this media (Figure 6B) shows a net increase in the levels of all vitamins except B1 supporting the conclusion about the availability of B-vitamins in the shared extracellular pool, which in the case of *in vitro* cultures could originate only from respective prototrophs. This effect appears to be masked for B1 by its unexpected presence, albeit in relatively low quantities, in the fresh AD media. Not surprisingly, the quantities of all four B-vitamins in the cell pellets (Figure 6C) and supernatants (Figure 6B) of samples grown in AN and AE media showed no significant difference compared to the respective fresh media. These levels were, however, substantially higher than samples grown under vitamin deficiency. Taken together with unaltered phenotype profiles (Figure 5), these results are consistent with the conclusion that B-vitamins are not limiting nutrients for auxotrophic species in these microbial communities under the tested growth conditions.

## B-Vitamin Sharing *In Vitro* Using *E. Coli* K-12 Knockout Strains

**FIGURE 7:**
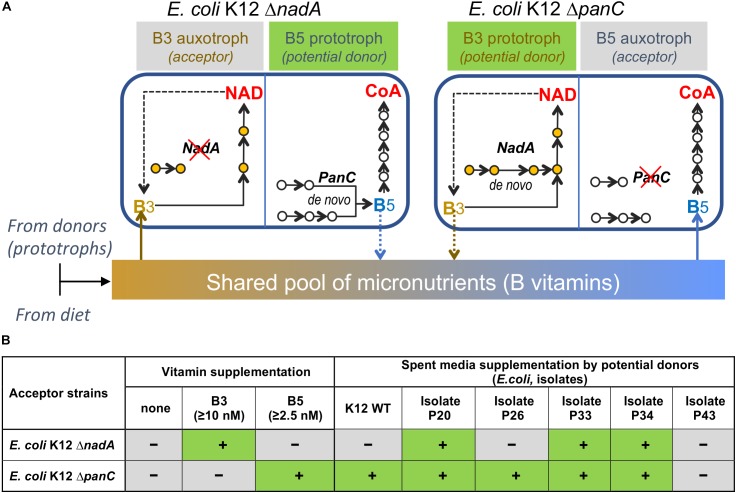
Modeling vitamin sharing *in vitro*. **(A)**. B-vitamin sharing hypothesis illustrated by the example of two *E. coli* K-12 knockout strains, B3 (ΔNadA) and B5 (ΔPanC) auxotrophs. **(B)**. Summary of growth rescue studies using the same *E. coli* K12 ΔNadA and ΔPanC tester strains as auxotrophic acceptors in B-vitamin-free liquid media supplemented by respective vitamins or supernatants from the cultures of *E. coli*, wild-type (K12-WT) or selected isolates from human fecal samples (Figure 1 and Supplementary Figure S1A for additional data).

To assess feasibility of vitamin exchange in a simple *in vitro* model, we used two knockout (KO) strains Δ*nadA* (B3 auxotroph) and Δ*panC* (B5 auxotroph) from the Keio *E. coli* mutant collection (Baba et al., 2006) as acceptors of respective vitamins and Δ*uxaC* strain (prototroph for both vitamins) as a potential donor (Figure 7A). The quantities of exogenous B-vitamin required to rescue and support maximal growth density of Δ*nadA* and Δ*panC* strains in M9 media are relatively low for both strains (2.5 nM and 10 nM, respectively (Supplementary Figure S6A). The ability of a prototroph to rescue the growth of these auxotrophs was tested by two methods: (i) by streaking Δ*nadA* and Δ*panC* strains side-by-side with Δ*uxaC* strain on M9 plates lacking B3 and B5; and (ii) by cultivating auxotrophic strains Δ*nadA* or Δ*panC* in liquid media lacking B3 and B5 and supplemented by supernatants of a prototrophic Δ*uxaC* culture grown in the same media for 6 h at 37°C. Both tests resulted in successful rescue of Δ*panC* but not Δ*nadA* strain (Supplementary Figure S6B), which may reflect relatively higher B3 requirement of Δ*nadA* strain and/or lower B3 sharing capacity of the prototrophic Δ*uxaC* strain. A potential strain-dependent ability of wild-type *E. coli* to function as B-vitamin donors was further tested using multiple *E. coli* isolates selected using CDM without B3 or B5 (B3 and B5 prototrophs) from human fecal samples. Surprisingly, the sequencing of full-length 16S rRNA revealed that the great majority of isolates recovered on solid media lacking B3 and B5 were *E. coli*, despite the fact that this species was not observed in appreciable abundance in the fecal inoculum (Supplementary Table S1F). We speculate that the selection procedure particularly the colony isolation step on solid media may have favored the recovery of this taxonomic group. The latter (liquid culture-based) approach to growth supplementation, showed that while most isolates tested (∼90) displayed some rescue capacity, the efficiency of growth rescue of either auxotroph varied significantly among the tested isolates (Figure 7B and Supplementary Figures S6C,D) suggesting that B-vitamin prototroph sharing capability in this proof of concept experiment is a quantitative trait controlled by yet unknown regulatory mechanisms.

## Discussion

This study of syntrophic metabolism of B-vitamins was to a large extent inspired by groundbreaking work by Goodman et al. (2009) which provided the first strong evidence of syntrophy in metabolism of vitamin B12 in the gut microbial community. Here, we extend the range of tested vitamins focusing mostly on those B-vitamins (B1, B2, B3, and B5) that (unlike B12) need to undergo a multi-step metabolic transformation upon uptake to yield functionally active essential cofactors. Therefore, the respective auxotrophs may be considered as having partial (downstream) pathways involved in “distributed” (or syntrophic) biogenesis of respective essential cofactors (TPP, FMN/FAD, NAD/NADP, and CoA). The analysis of multiple B-vitamin auxotrophies allowed us to define a widespread form of syntrophy as a basis of mutualism in host-associated microbial communities.

We demonstrate that feeding humanized gnotobiotic mice diets lacking all or some B-vitamins does not alter the fractional representation of B1, B2, B3, B5, B6, B7, B9 or B12 auxotrophic species (including numerous multi-auxotrophs). Our *in vivo* and *in vitro* model studies of human gut microbial communities showed that the fitness of B-vitamin auxotrophs is unaltered by B-vitamin depleted or replete growth conditions. This result strongly supports our hypothesis that the maintenance of B-vitamin auxotrophic segment of microbial populations occurs *via* community-wide B-vitamin sharing through as yet unknown mechanisms. These studies do not allow us to unambiguously state to what extent the observed vitamin sharing is driven by genuine excretion vs. cell lysis, we speculate that at least some B-vitamin prototrophs possess a general capacity to secrete B-vitamins into the extracellular milieu that are subsequently taken up by auxotrophic species thus ensuring their maintenance in the community.

We also tested whether the excess of B-vitamins exerts a selective pressure that favors B-vitamin auxotrophs whose fitness is no longer constrained by B-vitamin limitations and competition for these key substrates. The provision of excess B-vitamins did not impact the frequency of auxotrophs in the gut microbiota *in vitro* or *in vivo.* Taken together, these results provide an impressive example of microbiota stability in the face of extreme essential nutrient deprivation or excess. We speculate that in the absence of B-vitamin sharing, microbiota would be unable to harbor stable communities featuring auxotrophic species. In the event of periods of fasting or dietary limitations that may have been commonplace throughout evolutionary history, may have selected for B-vitamin sharing to maintain stable communities and functionally interacting taxa. It is likely that the evolution of B-vitamin sharing preceded the widespread loss of genes encoding B-vitamin biosynthetic capacity, thereby enabling gene loss of these pathways. A sizeable representation of auxotrophs (at least 20–30% by abundance) may reflect that B-vitamin sharing is highly efficient. These results are consistent with the black queen hypothesis that posits that fitness gain accompanying gene loss is frequency dependent, demanding the B-vitamin donors must remain in sufficient abundance in communities to ensure that auxotrophs are not subject to negative selection (Morris et al., 2012). It will be of interest to evaluate this further by colonization of GF mice with defined communities comprised of B-vitamin prototrophs/auxotrophs in various proportions under vitamin restricted diets to more directly establish the dependency of B-vitamin auxotrophs on adequate abundance of prototrophs.

Our proof of concept studies on growth supplementation of *E. coli* tester strains (B3 and B5 auxotrophs), illustrates B-vitamin sharing, in a simple model system that not all prototrophs (and/or not in all conditions) display uniform efficiency as B-vitamin donors (Supplementary Figure S6C). Further studies are required to elucidate genomic features (e.g., a particular kind of vitamin-sharing transporters as well as regulatory and physiological/biochemical factors) defining this capability across major prototrophs in gut microbial communities. Notably, the observed higher propensity for B5 vs. B3 sharing may reflect a fundamental difference in pathway topology. Indeed, pantothenate (B5) is a true intermediate in Coenzyme A biosynthesis, whereas niacin (B3) is a degradation product of the cofactor, NAD (Figure 7A). Therefore, one would expect the extent of NAD degradation and, thus, niacin accumulation (followed by recycling and/or excretion) to vary between strains and growth conditions.

It is important to note that the extreme stability was observed in this study for a particular microbial consortium with relatively low level of auxotrophy for nearly all B-vitamins. Without further studies, it may not be generalized to other gut microbial consortia, e.g., with a relatively high content of B-vitamin auxotrophs, which, based on our analysis of numerous HMP samples (Rodionov et al., 2019), is not uncommon. The latter notion may be illustrated by the comparison of B-vitamin auxotrophy levels observed in both *in vivo* and *in vitro* microbial communities (over the entire range of B-vitamin levels in tested diets/media) with a much wider distribution of the same phenotypes over 313 analyzed human fecal samples from HMP dataset (Figure 4).

The established extreme stability of gut microbiota in the face of extremes of B-vitamin bioavailability represent an important aspect of metabolite sharing occurring in host-associated microbial communities. This type of cooperative mutualism is likely to be commonplace in gut communities, which extends beyond B-vitamins *per se*, and may be viewed as a component of a large network of cooperative interactions involving cross-feeding and sharing of key metabolites. These interactions enable and highlight the capacity of bacterial communities to carry out complex biochemical transformations exceeding the capacity of its individual components. This study combined *in silico* phenotype predictions with experimental analysis of gut microbial communities, in this case focused on B-vitamin sharing. The curation and reconstruction of additional biochemical pathways encoded in gut resident genomes is highly expandable and may be effectively applied to a wide variety of studies focused on diet-microbiota, microbe-microbe and microbiota-host interactions.

B-vitamin auxotrophs are not limited to gut microbiota and exist in all human-associated microbiomes. An open question of interest relates to the efficiency of B-vitamin sharing in other domains of the human body, e.g., skin, where lower density communities and constrained diffusion of secreted factors may hamper cross-feeding phenomena. It also remains unclear whether B-vitamin donor phenotypes are broadly shared among prototrophs or a community service provided by subset of the community. Identification of gene functions that secrete B-vitamins is expected to greatly enhance our ability to address this open question. The evolution of B-vitamin auxotrophy in gut microbiota is thought to be the result of consistent B-vitamin availability from diet and/or microbial sources, permitting B-vitamin pathway gene shedding to occur. In the context of the studies presented here, we may further speculate that the overall “fitness” of microbiota is enhanced by an untold number of functional interactions and metabolic dependencies that make the whole greater than the sum of its parts. In the absence of mechanisms to share B-vitamins, communities, even in short-term deprivation of B-vitamin availability would be expected to result in the loss of auxotrophic species, resulting in the large-scale disruption of functional interactions occurring in a well-balanced community.

## Ethics Statement

All mouse experiments were performed using protocols approved by Johns Hopkins University Animal Care and Use Committee (IACUC number MO14M345).

## Author Contributions

SP and DP conceived and designed the research project. DP and HD performed the animal studies. VS and DT performed *in vitro* studies and DNA extraction for sequence analysis. DR, SL, and AO performed metabolic reconstructions and phenotype predictions. SI performed phylotype-to-phenotype computational analysis. SP and AO wrote the manuscript. All authors read and approved the final manuscript.

## Conflict of Interest Statement

The authors declare that the research was conducted in the absence of any commercial or financial relationships that could be construed as a potential conflict of interest.

## Footnotes

1 www.phenobiome.com
