# Supplementary material for: B-Vitamin Sharing Promotes Stability of Gut Microbial Communities
Source: Front Microbiol. 2019 Jul 2;10:1485. doi: 10.3389/fmicb.2019.01485 (PMC6615432; doi:10.3389/fmicb.2019.01485)
Supplement: Supplementary file 1 [file Data_Sheet_1.PDF]

Supplementary Figures: Legends

Figure S1. PCoA analysis of microbiota in extreme B vitamin diets. Bray-Curtis β-diversity of communities after 4 weeks on B vitamin diets. AD (green), AN (red), AE (blue*) in vivo* **(A) and *in vitro* (B)**

Figure S2. Comparison of phylogenetic profiles of microbial communities *in vivo* and *in vitro* vs HMP dataset. Dot plot representation of top 20 bacterial genera (by average relative abundance) in: **A.** fecal samples from colonized gnotobiotic mice aggregating all diets and timepoints (**Table S1A**); **B.** cell pellets from anaerobic cultures aggregating all tested growth media (**Table S2A); C.** in a subset of 313 fecal HMP samples

Figure S3. B vitamin auxotrophs in microbial communities from humanized gnotobiotic mice fed the diets supplemented with different levels of B vitamins. Examples of individual multi-auxotrophic species showing statistically significant increase in representation (relative abundance %) between a group of samples from three diets (AD, 4D4N and 7D1N) consistently deficient for vitamins B1, B2, B5 and B7 vs three other diets (AE, 4E4N and 7E1N), where the same subset of B vitamins is present in excessive amounts.

Figure S4. Comparison of B vitamin auxotrophy phenotype signatures of microbial communities cultured in vivo and *in vitro* with different levels of B vitamins. Predicted auxotrophy phenotypes signatures for each of the eight B vitamins computed for fecal samples from all individual diet groups of in vivo studies (Week 4) and for cell pellets from anaerobic cultures grown in defined media with different levels of B vitamins (**Table 1**) are shown by box plots.

Figure S5. B vitamin community phenotype signatures after renormalization by 16S rRNA copy numbers. (A) B vitamin auxotrophy phenotype signatures of in vivo and in vitro microbial communities vs HMP samples (a renormalized version of Figure 3); (B) Community-wide B-vitamin auxotrophy representation (%) in a series of *in vivo* samples representing three diets supplemented by water without B-vitamins (***AD***), with *normal* (***AN***) or *excessive* (***AE***) supplementation of B-vitamins (a renormalized version of **Figure 1**); (C) Same parameters determined for samples from anaerobic culturing grown under conditions of B-vitamin deficiency (***AD***), *normal* (***AN***) or *excessive* (***AE***) supply. (a renormalized version of Figure 4).

Figure S6. B vitamin sharing in vitro using E. coli K-12 knockout strains. A. Growth of *E. coli* K-12, Δ*nad*A and Δ*pan*C strains rescued by exogenous B vitamin in a dose-dependent manner. **B.** Δ*pan*C and Δ*nad*A growth in *uxa*C *E. coli* K12 conditioned media supernatants. **C and D.** Growth of Δ*nad*A (C) and Δ*pan*C (D) tester strains in conditioned media supernatants derived from representative human *E. coli* isolates.

Supplementary Figures

Figure S1

A **B**

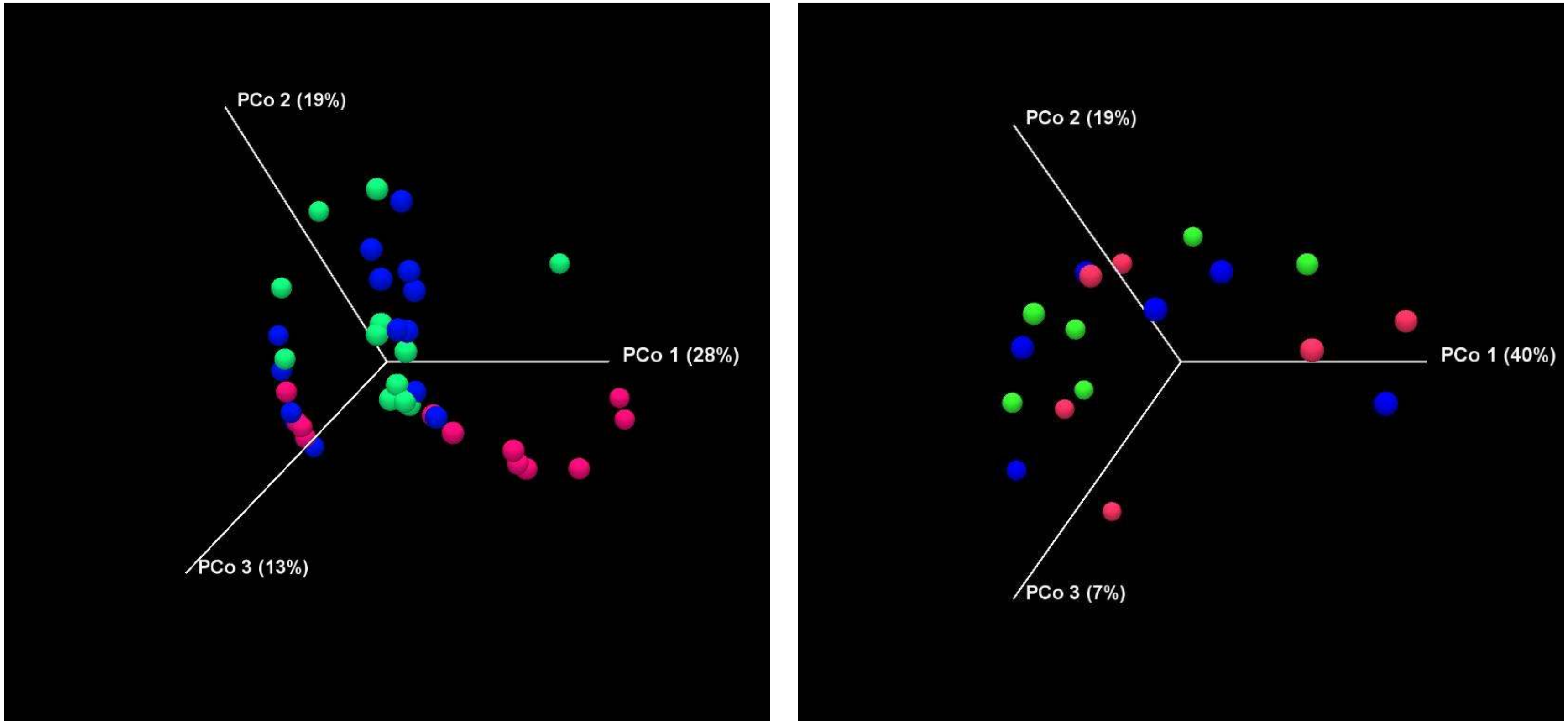

Figure S2

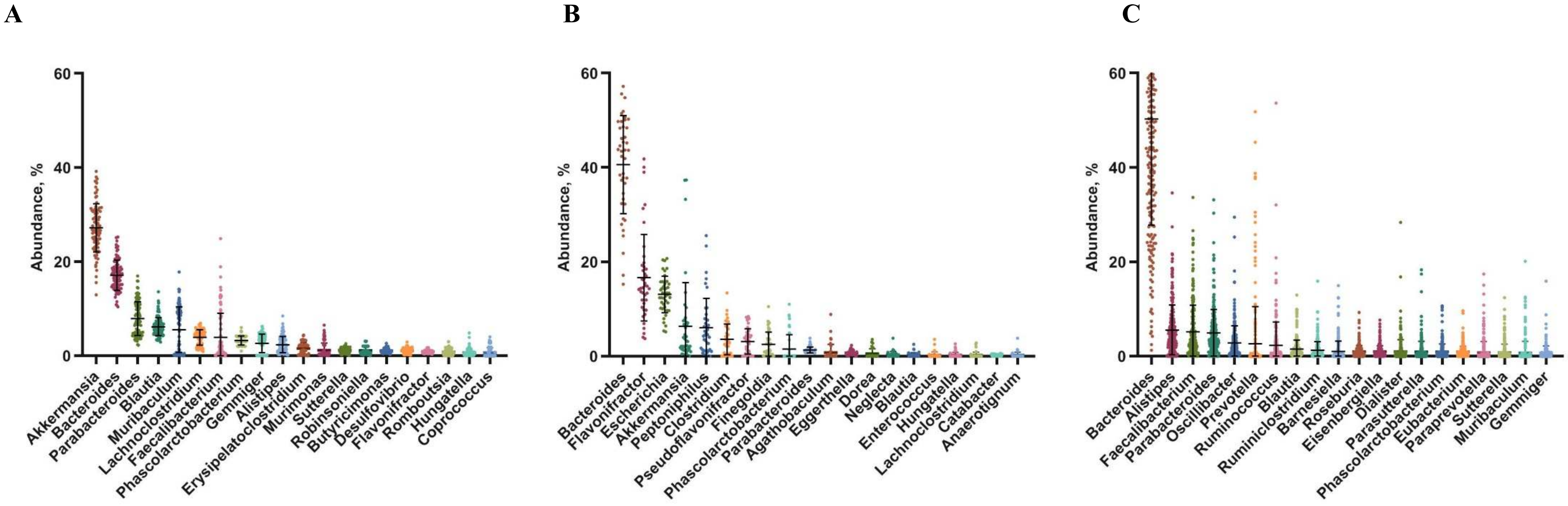

A
B
C
Abundance, %
0
20
40
60
Akkermansia
Bacteroides
Parabacteroides
Blautia
Muribaculum
Lachnoclostridium
Faecalibacterium
Phascolarctobacterium
Gemmiger
Alistipes
Erysipelatoclostridium
Murimonas
Sutterella
Robinsoniella
Butyricimonas
Desulfovibrio
Flavonifractor
Romboutsia
Hungatella
Coprococcus
Bacteroides
Flavonifractor
Escherichia
Akkermansia
Peptoniphilus
Clostridium
Pseudoflavonifractor
Finegoldia
Phascolarctobacterium
Parabacteroides
Agathobaculum
Eggerthella
Dorea
Neglecta
Blautia
Enterococcus
Hungatella
Lachnoclostridium
Catabacter
Anaerotignum
Bacteroides
Alistipes
Faecalibacterium
Parabacteroides
Oscillibacter
Prevotella
Ruminococcus
Blautia
Ruminiclostridium
Barnesiella
Roseburia
Eisenbergiella
Dialister
Parasutterella
Phascolarctobacterium
Eubacterium
Paraprevotella
Sutterella
Muribaculum
Gemmiger

Figure S3

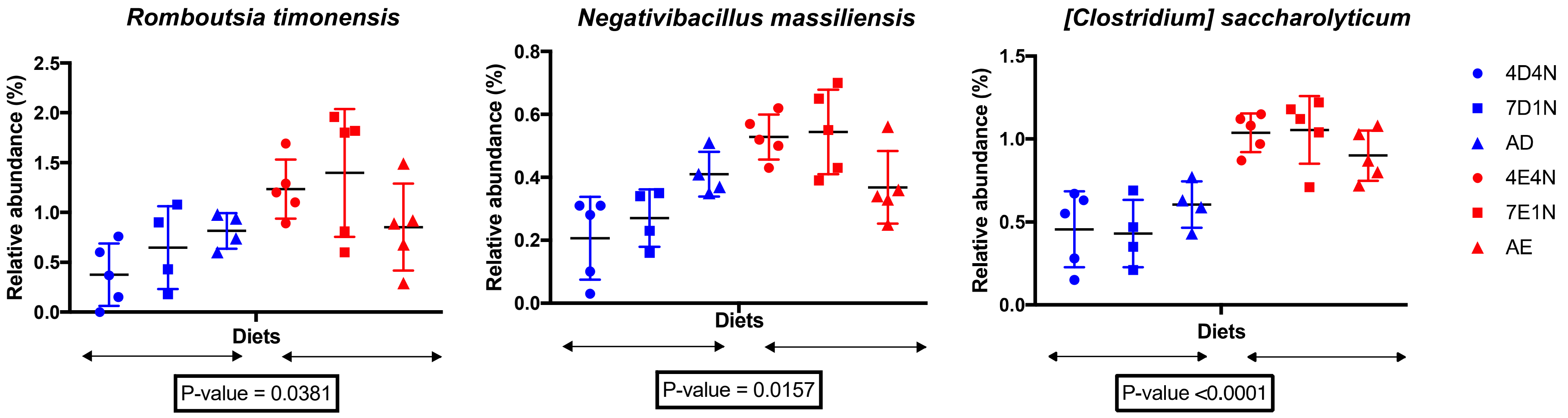
Romboutsia timonensis
Relative abundance (%)
Diets
P-value = 0.0381
Negativibacillus massiliensis
Relative abundance (%)
Diets
P-value = 0.0157
[Clostridium] saccharolyticum
Relative abundance (%)
Diets
P-value <0.0001
4D4N
7D1N
AD
4E4N
7E1N
AE

Figure S4.

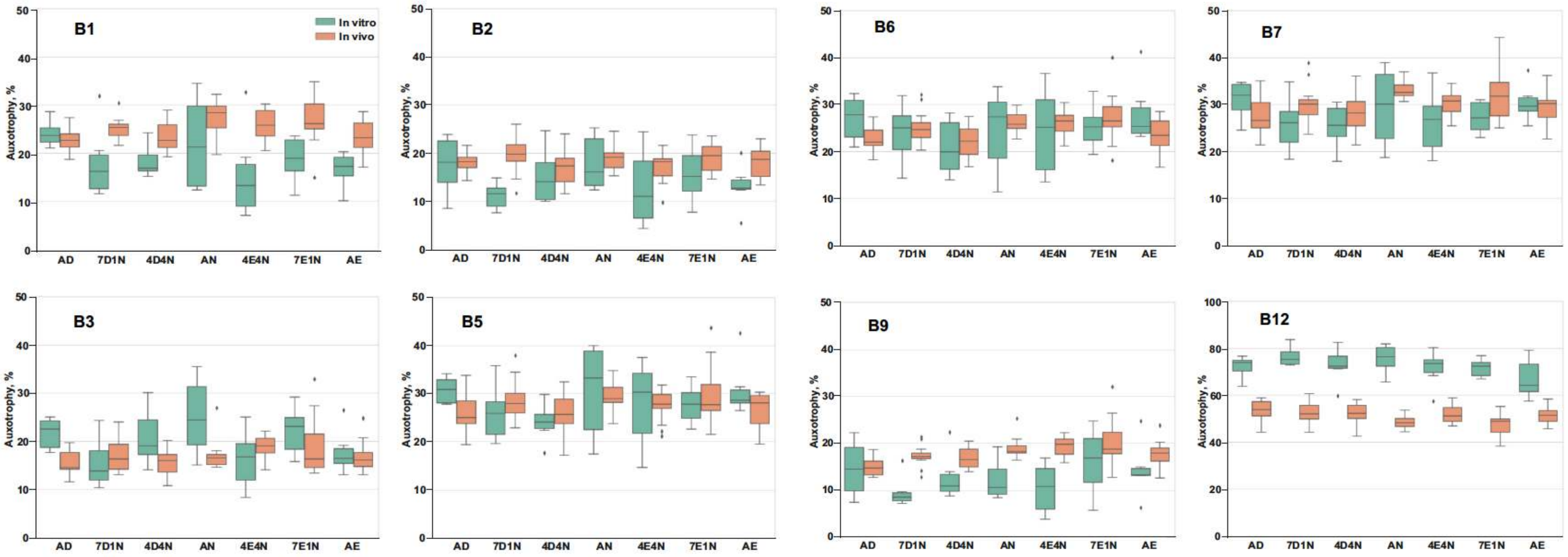
B1
B2
B6
B7
B3
B5
B9
B12
In vitro
In vivo
Auxotrophy, %
AD
7D1N
4D4N
AN
4E4N
7E1N
AE

Figure S5

A

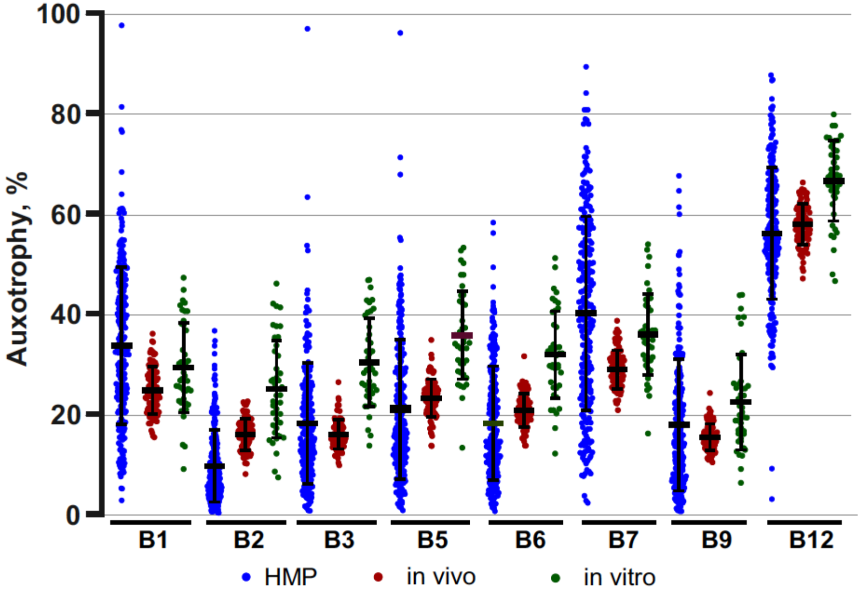
Auxotrophy, %
100
80
60
40
20
0
B1
B2
B3
B5
B6
B7
B9
B12
HMP
in vivo
in vitro

B

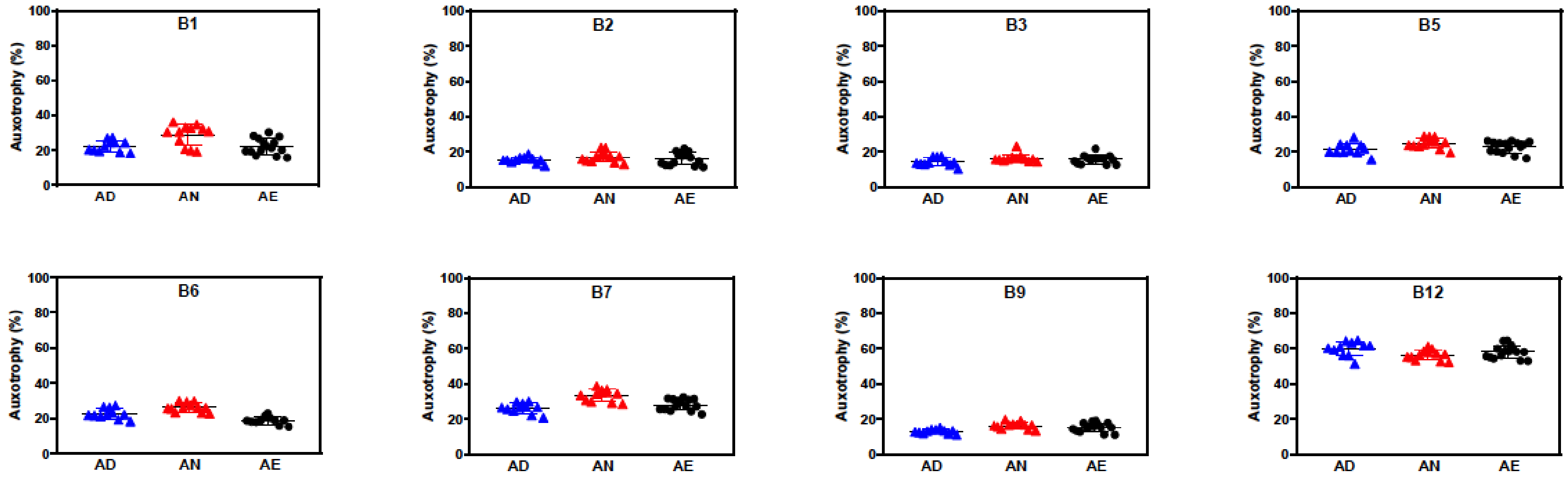
B1
B2
B3
B5
B6
B7
B9
B12
Auxotrophy (%)
AD
AN
AE

C

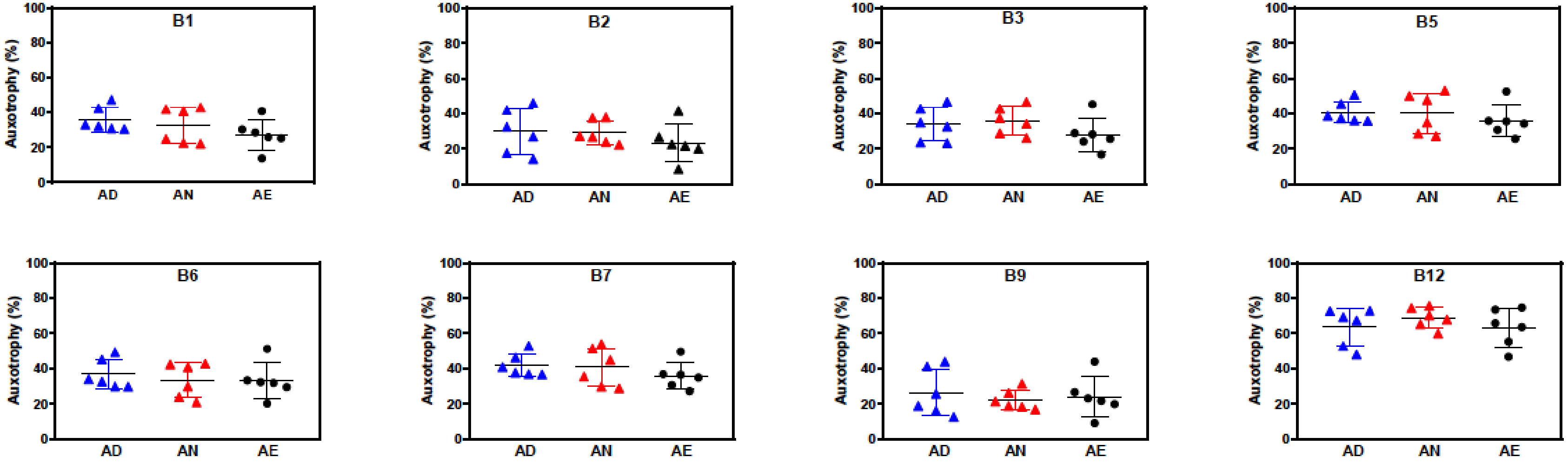
B1
B2
B3
B5
B6
B7
B9
B12
Auxotrophy (%)
AD
AN
AE

Figure S6

A

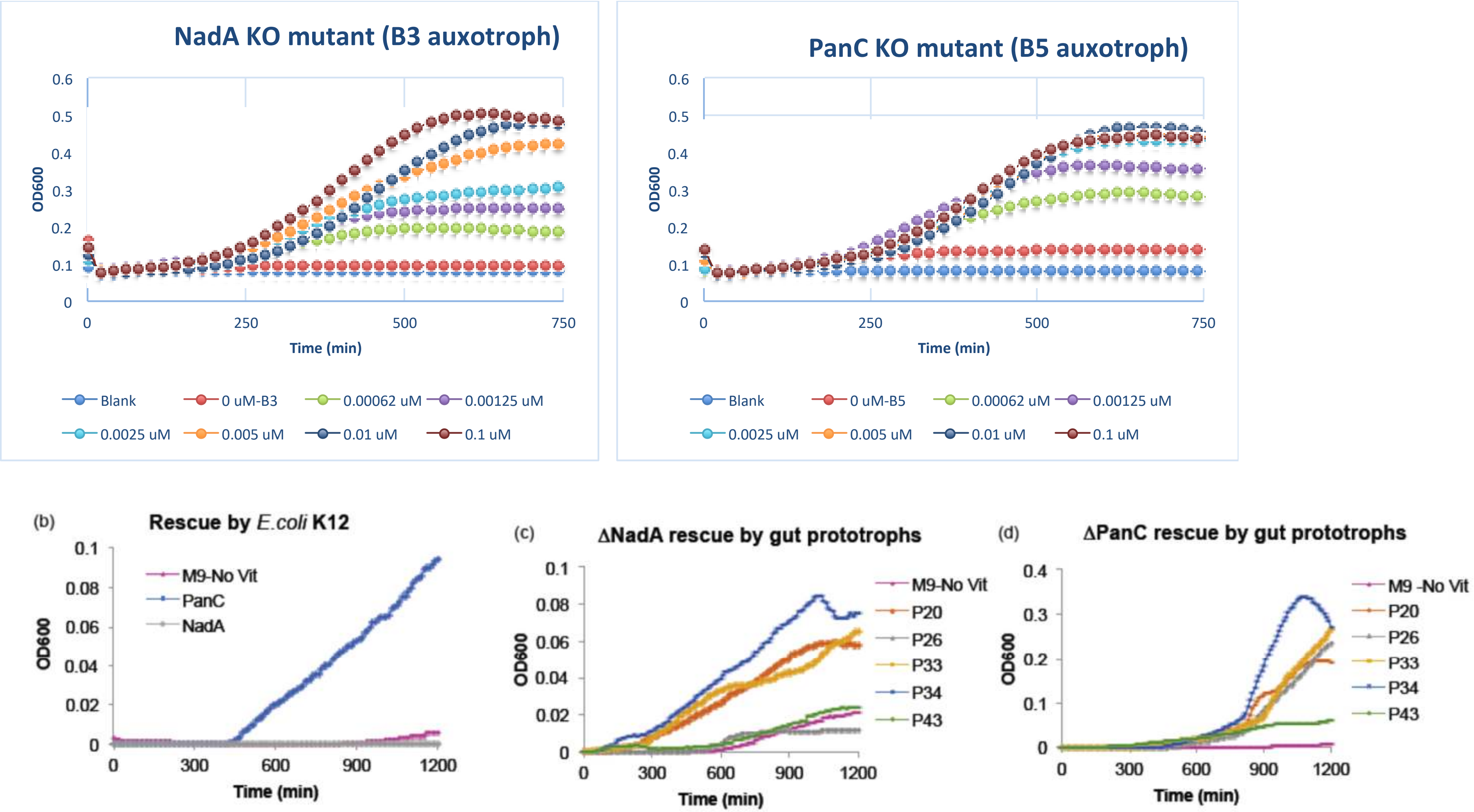
NadA KO mutant (B3 auxotroph)
OD600
Time (min)
Blank
0 uM-B3
0.00062 uM
0.00125 uM
0.0025 uM
0.005 uM
0.01 uM
0.1 uM
PanC KO mutant (B5 auxotroph)
OD600
Time (min)
Blank
0 uM-B5
0.00062 uM
0.00125 uM
0.0025 uM
0.005 uM
0.01 uM
0.1 uM
(b)
Rescue by E.coli K12
OD600
Time (min)
M9-No Vit
PanC
NadA
(c)
ΔNadA rescue by gut prototrophs
OD600
Time (min)
M9-No Vit
P20
P26
P33
P34
P43
(d)
ΔPanC rescue by gut prototrophs
OD600
Time (min)
M9 -No Vit
P20
P26
P33
P34
P43
